# Nordamnacanthal potentiates the cytotoxic effects of tamoxifen in human breast cancer cells

**DOI:** 10.3892/ol.2014.2697

**Published:** 2014-11-10

**Authors:** TAMILSELVAN SUBRAMANI, SWEE KEONG YEAP, WAN YANG HO, CHAI LING HO, CHE PUTEH OSMAN, NOR HADIANI ISMAIL, NIK MOHD AFIZAN NIK ABDUL RAHMAN, NOORJAHAN BANU ALITHEEN

**Affiliations:** 1 Department of Cell and Molecular Biology, Faculty of Biotechnology and Biomolecular Sciences, Universiti Putra Malaysia, Serdang, Selangor 43400, Malaysia; 2 Institute of Bioscience, Universiti Putra Malaysia, Serdang, Selangor 43400, Malaysia; 3 Faculty of Medicine and Health Science, School of Biomedical Sciences, The University of Nottingham Malaysia Campus, Semenyih, Selangor 43500, Malaysia; 4 Faculty of Applied Sciences, Universiti Teknologi Mara, Shah Alam, Selangor 40450, Malaysia

**Keywords:** tamoxifen, nordamnacanthal, MCF-7, breast cancer, combination treatment, apoptosis

## Abstract

Tamoxifen (TAM) is the mainline drug treatment for breast cancer, despite its side effects and the development of resistance. As an alternative approach, in the present study a novel combination therapy was established through combining TAM with nordamnacanthal (NDAM) in order to investigate the additive effect of these drugs in MCF-7 human breast cancer cells. A significant dose-dependent reduction in cell viability and an increase in apoptosis were observed in the MCF-7 cells cotreated with TAM and NDAM compared with the untreated control cells or the cells treated with TAM and NDAM alone (P*<*0*.*05). The cytotoxic influence of the combination of TAM and NDAM was found to be two-fold that of the individual agents. Annexin V/propidium iodide double-staining revealed the typical nuclear features of apoptosis. Furthermore, an increase in the proportion of apoptotic, Annexin V-positive cells was observed with the combination therapy. Moreover, this apoptotic induction was associated with a collapse of the mitochondrial membrane potential and the generation of reactive oxygen species. To the best of our knowledge, the findings of the present study are the first to suggest that combining TAM with NDAM may be a potential combination therapy for the treatment of breast cancer and may have the potential to minimize or eliminate the side effects associated with high doses of TAM.

## Introduction

Breast cancer is the most common type of cancer in the female population (1). Despite significant progress in the treatment of breast cancer over the past decades, breast cancer remains the primary cause of cancer-related mortality in females worldwide (1,2). The development and progression of this chronic disease involves the deregulation and activation of multiple signaling pathways at various stages in carcinogenesis. This complexity associated with breast cancer causes limitations in designing high-efficacy therapeutic strategies. Tamoxifen (TAM) is the mainline drug prescribed for patients with metastatic breast cancer (3). TAM has been reported to reduce the risk of recurrence and mortality in patients with breast cancer when administered as an adjuvant therapy (4). TAM is a non-steroidal selective estrogen receptor modulator, which is particularly effective in post-menopausal women who have a significant risk of developing estrogen receptor-positive breast cancer (5,6). The antitumor activity of TAM has been proposed to be cytostatic and cytotoxic for breast cancer. More specifically, TAM has been shown to interact with the mitochondrial estrogen receptor and increase the reactive oxygen species concentrations from the mitochondria required for the cytotoxicity (7,8). However, there are serious side effects associated with the prolonged use of high-dose TAM, particularly in the uterus, which manifests as abnormal proliferation and an increase in the risk of endometrial cancer (9–12). Therefore, novel therapeutic approaches that could increase TAM sensitivity are required, so that lower doses may be used without compromising TAM efficacy. One potential strategy may be the combination of TAM with other agents that increase the efficacy and decrease the toxicity of TAM. Nordamnacanthal (NDAM), also known as 2-formyl-1,3-dihydroxyanthraquinone, is an anthraquinone extracted from the roots of *Morinda elliptica* (13). NDAM has been reported to have a number of biological properties, including antitumor effects (14,15). A previous study has shown that depending upon the concentration/dose, NDAM is capable of triggering and blocking cell death signaling in tumor cells (16). Since both TAM and NDAM have antitumor properties, a combination of these drugs may be a therapeutic option for patients with breast cancer. However, their potential additive effects have yet to be elucidated. Therefore, the present study aimed to investigate the effect of TAM and NDAM on apoptosis, cell cycle arrest, mitochondrial membrane potential (Δψ_m_) and oxidative stress in MCF-7 human breast cancer cells.

## Materials and methods

### Cells and cell culture

The estrogen-sensitive MCF-7 human breast cancer cell line was obtained from the American Type Culture Collection (Rockville, MD, USA). Cells were cultured as monolayers in RPMI-1640 (Sigma-Aldrich, , St. Louis, MO, USA) medium supplemented with 10% heat-inactivated fetal bovine serum (Gibco-BRL, Carlsbad, CA, USA), 100 U/ml penicillin and 100 μg/ml streptomycin (both Sigma-Aldrich) at 37°C in a humidified environment containing 5% CO_2_.

### Drugs and drug treatment

**Figure 1:**
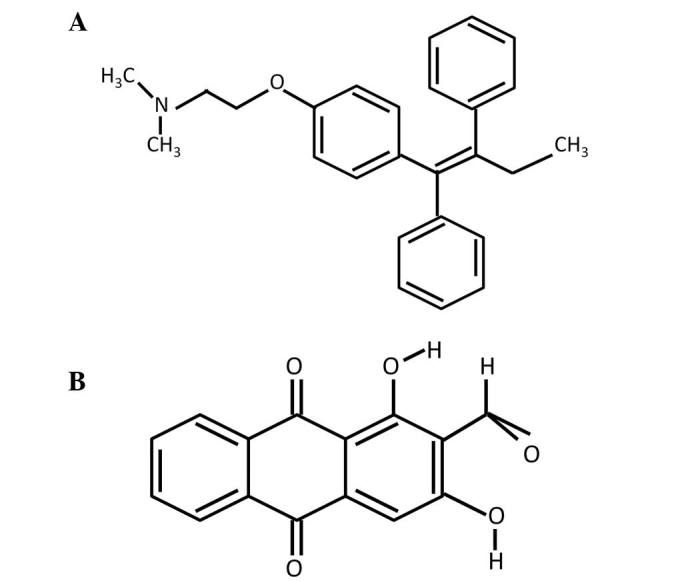
Chemical structure of (A) tamoxifen and (B) nordamnacanthal.

NDAM was isolated from the roots of *M. elliptica* using solvent fractionation and was purified using high-performance liquid chromatography techniques. The structure was identified by comparing spectroscopic data as reported in our previous study (13). NDAM was dissolved in dimethyl sulfoxide (DMSO; Sigma-Aldrich) and stored at −20°C as a 10-mg/ml stock solution. TAM (Sigma-Aldrich) was dissolved in DMSO at a concentration of 10 mg/ml. In the control experiments, equal quantities of DMSO were added. The structure of TAM and NDAM are shown in Fig. 1.

### Cytotoxicity assay

Cells were seeded at a density of 5000 cells/well in 96-well microtiter culture plates and incubated overnight. The culture medium was replaced with fresh medium containing various concentrations of NDAM (0–30 μg/ml) and TAM (0–30 μg/ml) alone or in combination for 24, 48 and 72 h. A total of 20 μl 3-(4, 5-dimethylthaiazol-2-yl)-2-5-diphenyltetrazolium bromide (MTT; Calbiochem, Darmstadt, Germany) solution (0.5 mg/ml) was added to each well and cell proliferation was analyzed as described previously (14).

### Cell viability assay

Cell death was quantified in the MCF-7 cells using propidium iodide (PI; Sigma-Aldrich) and acridine orange (AO; Sigma-Aldrich) double-staining as described previously, using a fluorescence microscope (Diaphot, Nikon Inc., Melville, NY, USA) (14). The MCF-7 cells were seeded in a 25-ml culture flask at a concentration of 1×10^6^ cells/ml and treated with NDAM and TAM alone or in combination and were incubated at 37°C with 5% CO_2_ for 72 h. The cells were washed with phosphate-buffered saline (PBS) and stained with 10 μl AO (10 μg/ml) and PI (10 μg/ml). Slides were analyzed under UV-fluorescence microscopy (Nikon Inc.) and the number of viable, apoptotic and necrotic cells was calculated.

### Annexin V binding assay

MCF-7 cells were treated with NDAM and TAM alone or in combination for 72 h. Cells were resuspended in Annexin V binding buffer (BD Pharmingen, San Diego, CA, USA) to a concentration of 1×10^6^ cells/ml. Annexin V-fluorescein isothiocyanate (FITC; BD Biosciences, San Diego, CA, USA) was incubated for 15 min in the dark in 100 μl cell suspension. PI was then spiked into 400 μl Annexin V binding buffer and added immediately to the cell suspension, and subsequently analyzed using a FACSCaliber™ system with CellQuest™ software (both BD Biosciences). Care was taken to collect trypsinized cells and cells which may have been floating prior to trypsinization to ensure that apoptotic cells, if present, were detected.

### Cell cycle analysis

MCF-7 cells (5,000 cells/ml) were seeded onto 25 cm^2^ flasks with NDAM and TAM either alone or combined for 72 h. The cells were trypsinized and washed with PBS then centrifuged at 2,000 × g for 5 min. The cell pellet was resuspended in 1 ml 0.1% sodium citrate containing 0.05 mg PI and 50 μg RNase (Sigma-Aldrich) for 30 min at room temperature in the dark. Flow cytometric analysis was performed using a FACScan system (BD Biosciences) and CellQuest software.

### Assessment of Δψ_m_

MCF-7 cells (1×10^6^) were grown in 25-ml culture flasks for 24 h, followed by incubation with NDAM and TAM alone or in combination in culture medium for 72 h at 37°C. The Δψ_m_ was assessed using a BD™ MitoScreen kit (BD Biosciences) according to the manufacturer’s instructions, and analyzed using a FACSCaliber system (BD Biosciences). The ratio of Δψ_m_/mitochondrial mass was calculated to correct the Δψ_m_ for differences in mitochondrial mass.

### Assessment of lipid peroxidation

Lipid peroxidation was assessed through analyzing the lipid peroxidation marker malondialdehyde in the cell lysates (17,18). Cells were treated with NDAM and TAM alone or in combination at 37°C with 5% CO_2_ for 72 h. The treated MCF-7 cells were washed in ice-cold PBS and lysed in 260 μl solubilization buffer [10 mM Tris (pH 7.4), 9 g/l NP40, 1 g/l SDS and 250 U/ml benzonase; Sigma-Aldrich). Approximately 200 μl cell lysate or malondialdehyde standards (Sigma-Aldrich) were mixed with 10 μl butylated hydroxytoluene (50 mg/ml ethanol) and 200 μl orthophosphoric acid (0.2 mM). The reaction mixture was incubated on ice for 30 min and centrifuged at 2000 × g for 15 min at 25°C. The supernatant was separated and added to 25 μl 2-thiobarbituric acid reagent (800 mg 2-thiobarbituric acid dissolved in 50 ml 0.1 M NaOH) and incubated at 90°C for 45 min. Formed malondialdehyde equivalents, thiobarbituric acid-reactive substances (TBARS), were extracted and measured using a plate reader (Bio-Rad Laboratories, Inc.) with excitation at 532 nm and 600 nm. Malondialdehyde standard solution was used for qualitative determination of TBARS. The Bradford assay was performed in order to measure the protein content.

### Statistical analysis

All experiments were performed in triplicate. The statistical significance of the differences was determined using one-way analysis of variance followed by Dunnett’s multiple comparison test. Values are presented as the mean ± standard deviation and P<0.05 was considered to indicate a statistically significant difference.

## Results

### NDAM enhances the cytotoxic effect of TAM

**Figure 2:**
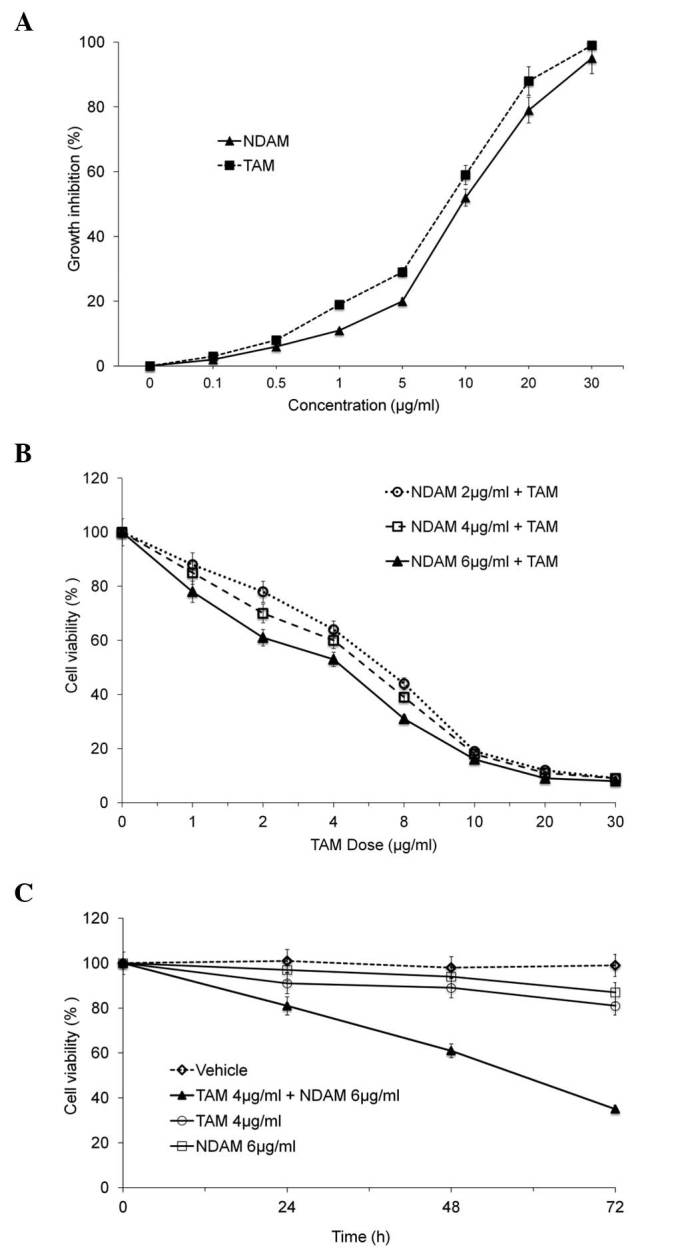
MCF-7 proliferation following treatment with TAM and NDAM alone or combined. Cells growing in the log-phase were trypsinized and plated onto 96-well plates at a density of 5,000 cells/well. Following incubation for 24 h, cells were treated with various concentrations of TAM and NDAM either (A) alone or (B) combined. (C) After 24, 48 and 72 h of treatment, cell proliferation was assessed using 3-(4, 5-dimethylthaiazol-2-yl)-2–5-diphenyltetrazolium bromide assay. Data are presented as the mean ± standard deviation of three independent experiments. TAM, tamoxifen; NDAM, nordamnacanthal.

In the present study, it was hypothesized that TAM in combination with NDAM may be a superior therapeutic strategy for breast cancer. In order to test this hypothesis, the effect of incremental doses of TAM and NDAM, alone or combined, was analyzed on the growth of MCF-7 human breast cancer cells. Cellular growth, determined using MTT assay, revealed that TAM and/or NDAM were effective in inhibiting the growth of the MCF-7 cells in a dose-dependent manner (Fig. 2A). Low doses of TAM (4 μg/ml) were observed to reduce the proliferation of breast cancer cells and complete cell death was achieved at a concentration of 23 μg/ml following treatment for 72 h (Fig. 2B). At a concentration of 6 μg/ml, NDAM was found to reduce cell viability by 24.5% and complete cell death was achieved at a concentration of 28 μg/ml NDAM (Fig. 2B). Upon combining NDAM with TAM, a markedly enhanced induction of cell death was observed, even at lower TAM concentrations (Fig. 2B). While treatment with 4 μg/ml TAM alone did not significantly reduce MCF-7 cell viability (12%), the combination of NDAM (6 μg/ml) and TAM (4 μg/ml) was found to significantly reduce MCF-7 cell viability by up to 77.0% (Fig. 2C; P<0.05). The same concentrations of TAM and NDAM were not sufficient to induce apoptosis when used alone.

### NDAM enhances TAM-induced apoptosis

**Figure 3:**
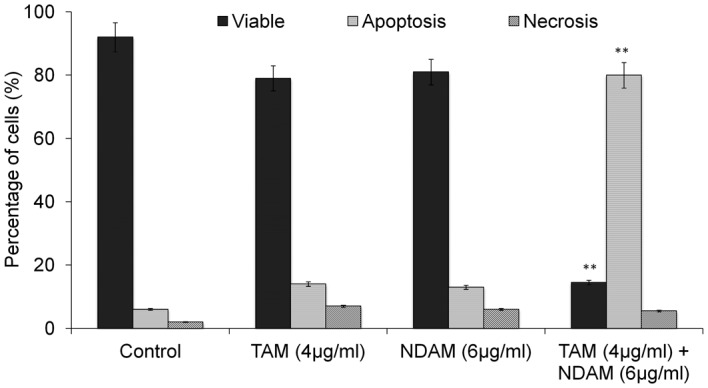
Morphological assessment of MCF-7 cells stained with acridine orange and propidium iodine following treatment with TAM and NDAM alone or combined. Cells were incubated with TAM (4 μg/ml) or NDAM (6 μg/ml) or a combination of TAM and NDAM for 72 h. Data are presented as the mean ± standard deviation of three independent experiments. ^**^P<0.05 vs. control. TAM, tamoxifen; NDAM, nordamnacanthal.

**Figure 4:**
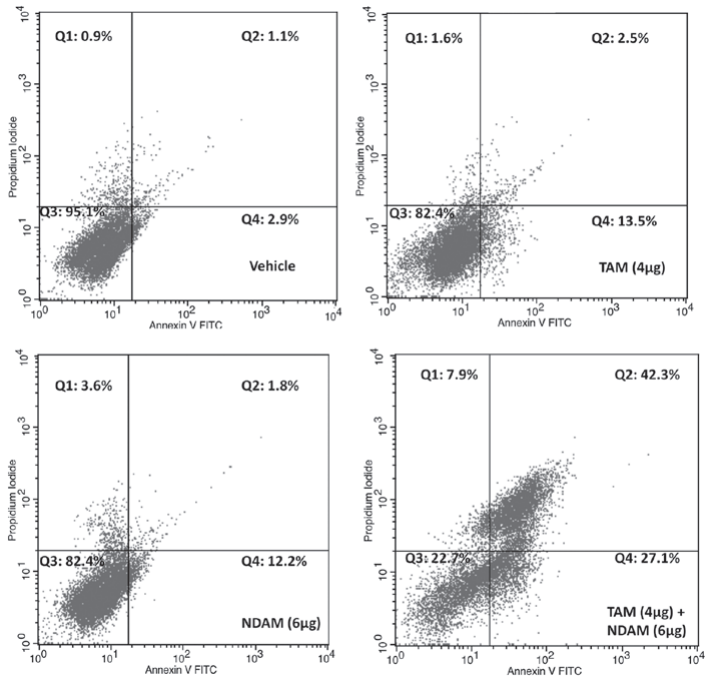
Flow cytometric analysis of MCF-7 cells treated with TAM (4 μg/ml) and NDAM (6 μg/ml) alone or in combination for 72 h and stained with Annexin V-FITC/propidium iodide. Graphs are representative of 10,000 cells from a single replicate. TAM, tamoxifen; NDAM, nordamnacanthal; FITC, Fluorescein isothiocyanate.

**Figure 5:**
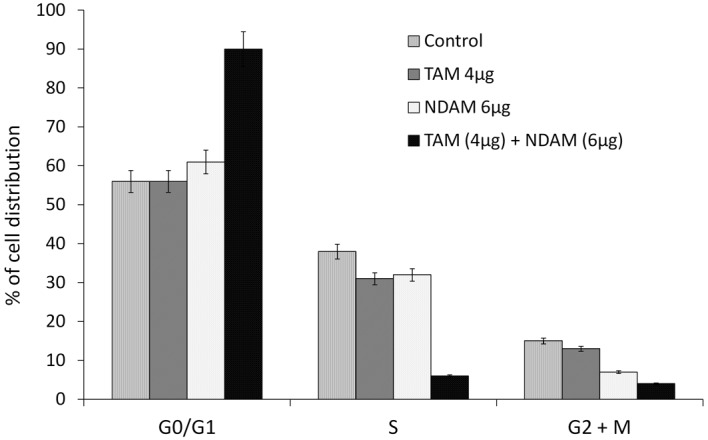
Cell cycle analysis detected using flow cytometry. MCF-7 cells treated with TAM (4 μg/ml) and NDAM (6 μg/ml) either alone and in combination for 72 h were stained with propidium iodide. The control cells were treated with dimethyl sulfoxide. Results are representative of three independent experiments. Data are presented as the mean ± standard deviation (n=4) of the percentage of cells in individual phases of the cell cycle from four independent experiments. TAM, tamoxifen; NDAM, nordamnacanthal.

Morphological changes were observed in the cells treated with the TAM/NDAM combination. Co-incubation of MCF-7 cells with TAM and NDAM resulted in significantly increased levels of apoptosis (Fig. 3) compared with the control cells and those treated with TAM or NDAM alone, with the proportion of the viable cells accounting for only 14.5% of the total cell population and 79% undergoing apoptosis. Necrotic cells were also observed in the treatment group, but the numbers were insignificant. Treatment with NDAM (6 μg/ml) and TAM (4 μg/ml) alone was not found to induce significant levels of apoptosis in the MCF-7 cells (Fig. 4). Annexin V-FITC analysis was performed to confirm the induction of apoptosis in the MCF-7 cells upon cotreatment with TAM and NDAM. Fluorescence-activated cell sorting revealed a significant increase in apoptotic cells (69%) upon cotreatment with TAM and NDAM for 72 h compared with the vehicle-treated cells (4%) or those treated with TAM (16%) or NDAM (14%) alone (Fig. 4). Cell cycle analysis revealed an increase in G_0_/G_1_-phase accumulation in the TAM/NDAM-treated cells (63%; P<0.01) compared with the DMSO-treated cells (45%), with a concomitant decrease in the percentage of cells in the G_2_/M phase observed in the TAM/NDAM-treated cells (Fig. 5). Cell cycle arrest occurred from 24 h of treatment (data not shown) and longer treatment durations showed that TAM/NDAM induced G_0_/G_1_ arrest and apoptosis in the treated cells. However, no significant increases in cell cycle arrest were observed in the MCF-7 cells treated with NDAM (6 μg/ml) and TAM (4 μg/ml) alone.

### NDAM enhances the TAM-induced changes in Δψ_m_ and lipid peroxidation

**Figure 6:**
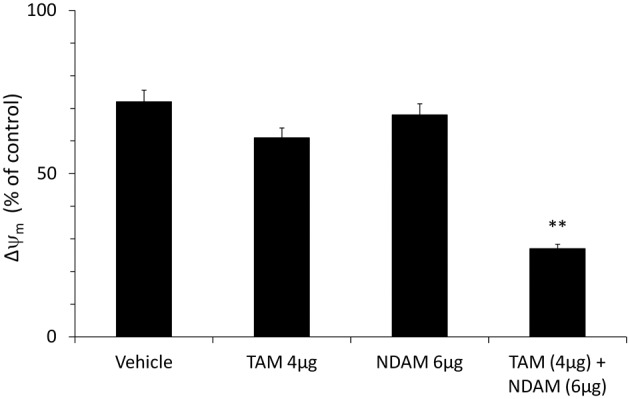
MCF-7 cells exposed to TAM (4 μg/ml) and NDAM (6 μg/ml) alone or in combination for 72 h. The Δψ_m_ was determined using flow cytometry. The Δψ_m_ of the MCF-7 cells treated with TAM/NDAM decreased significantly compared with that of the control cells and those treated with TAM or NDAM alone. Δψ_m_ levels, expressed as mean fluorescence intensity, were calculated as a percentage of the control. Data are presented as the mean ± standard deviation of at least triplicate experiments. ^**^P<0.05 vs. control. TAM, tamoxifen; NDAM, nordamnacanthal; Δψm, mitochondrial membrane potential.

**Figure 7:**
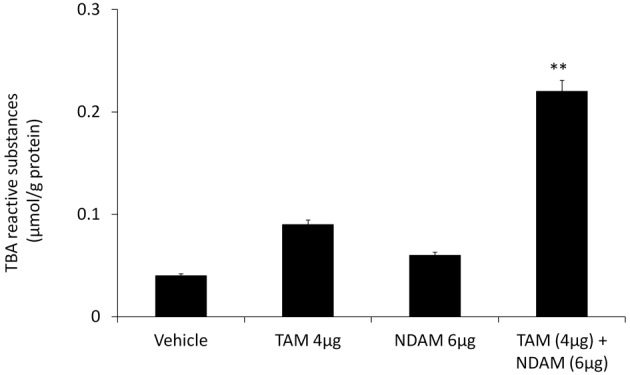
TBA reactive substances release from MCF-7 cells exposed to TAM (4 μg/ml) and NDAM (6 μg/ml) either alone or in combination for 72 h. Lipid peroxidation was analyzed through measuring the malonaldehyde equivalents TBA reactive substances. A significant increase in TBA reactive substances was observed in the MCF-7 cells following treatment with TAM/NDAM compared with that of treatment with TAM or NDAM alone. Data are presented as the mean ± standard deviation of at least three independent experiments.^**^P<0.05 vs. control. TBA, thiobarbituric acid; TAM, tamoxifen; NDAM, nordamnacanthal.

Changes in *Δ*ψ_m_ are considered to be an indicator of mitochondrial damage. It has also been reported that high quantities of reactive oxygen intermediates result in lipid peroxidation (18). Therefore, the present study analyzed the Δψ_m_ and the lipid peroxidation end product malondialdehyde in MCF-7 cells treated with TAM and NDAM, alone or combined. As shown in Fig. 6, changes in the Δψ_m_ were observed in the in MCF-7 cells following TAM/NDAM exposure for 72 h. By contrast, administration of NDAM (6 μg/ml) or TAM (4 μg/ml) alone had no significant effect on the Δψ_m_. The Δψ_m_ in the DMSO-treated cells was unchanged throughout all of the incubation time-periods. Furthermore, TAM/NDAM cotreatment was observed to have an effect on lipid peroxidation, as demonstrated by the significant release of the malondialdehyde equivalent TBARS from treated MCF-7 cells (Fig. 7). However, treatment with NDAM (6 μg/ml) and TAM (4 μg/ml) alone were not found to induce significant TBARS release in the MCF-7 cells.

## Discussion

TAM has been used for more than two decades for hormone therapy in breast carcinomas expressing the estrogen receptor. Although TAM is well tolerated and has resulted in a 5–15% absolute reduction in recurrence and mortality (4), more effective treatments for estrogen receptor-positive breast cancer are required. In addition to the side effects associated with TAM, it has been estimated that 90% of patients with breast cancer acquire resistance to TAM within one year (19). Support has increased for the use of natural compounds that enhance the therapeutic effect of antineoplastic agents so that lower doses may be used to achieve the same antineoplastic effect, while simultaneously avoiding or minimizing the side effects associated with high doses. In the present study, the results showed that the cytotoxic effect of TAM on breast cancer cells was enhanced through combined treatment with NDAM, thus allowing the effective concentration of TAM to be reduced. The growth inhibition advantage of combining NDAM with TAM was primarily due to enhanced cell cycle arrest and apoptosis. The cytotoxic effect was assessed through combining NDAM (6 μg/ml) at a concentration which did not enhance apoptosis, with the chemotherapeutic agent TAM (4 μg/ml). When NDAM was combined with a subapoptotic dose of the chemotherapeutic agent, significant apoptosis was induced. The primary growth inhibitory mechanism for TAM in MCF-7 cells has been reported to be cell cycle inhibition (20,21). In the present study, TAM/NDAM treatment was found to induce significant G_0_/G_1_-phase arrest in the MCF-7 cells. By contrast, treatment with TAM or NDAM did not induce a significantly greater G_0_/G_1_-phase arrests in the MCF-7 cells compared with TAM/NDAM cotreatment. Previous studies have shown that TAM induces significant G_0_/G_1_-phase arrest in breast cancer cells (20,22). Furthermore, in agreement with the present study, Li *et al* (23) showed that combined treatment of TAM with organoselenium compounds enhanced apoptosis. These results show that TAM/NDAM-induced cell growth inhibition is concomitant with major changes in the cell cycle in MCF-7 cells.

In mammalian cells, the mitochondria have a fundamental role in apoptosis. At the early stage of apoptosis, mitochondrial damage occurs through disruption of the Δψ_m_ which leads to the activation of caspase cascades (24). In the present study, the combination of TAM/NDAM was observed to induce a significant loss of Δψ_m_ in the MCF-7 breast cancer cells. However, when low doses of TAM and NDAM were applied individually, neither of the drugs caused damage to the mitochondrial membrane. Furthermore, in the present study, an increase in TBARS release was observed following TAM/NDAM treatment for 72 h, while no significant TBARS release was found following treatment with TAM or NDAM alone. These findings suggest that TAM/NDAM-induced TBARS release from MCF-7 cells may have caused the loss of Δψ_m_. In conclusion, in the present study, NDAM was found to enhance the cytotoxic activity of TAM, with the inhibition of cell proliferation, G_0_/G_1_-phase arrest, the generation of oxidative damage and the loss of Δψ_m_ cumulating in apoptosis following TAM/NDAM cotreatment. Combining NDAM with TAM was found to reduce the dose of TAM required to achieve the same therapeutic effect; therefore, this combination therapy has the potential to be a treatment regimen for breast cancer with minimal or no side effects that are frequently associated with high doses of TAM. However, it is important to investigate the safety and tolerability of TAM/NDAM *in vivo*.
